# Supplementary material for: From sequence to enzyme mechanism using multi-label machine learning
Source: BMC Bioinformatics. 2014 May 19;15:150. doi: 10.1186/1471-2105-15-150 (PMC4229970; doi:10.1186/1471-2105-15-150)
Supplement: Additional file 2 — Java code of ml2db. Additional file ml2db_code.tar.gz contains the Java source code to run the multi-label machine learning experiments and save the results to database. The code’s Javadoc is included. [file 1471-2105-15-150-S2.zip › additional file 2/ml2db/ml2db/doc/main/ml2db/al/utility/globaldistance/DistancesCalculator.html]

DistancesCalculator


JavaScript is disabled on your browser.


- Overview
- Package
- Class
- Use
- Tree
- Deprecated
- Index
- Help

- Prev Class
- Next Class

- Frames
- No Frames

- All Classes

- Summary:
- Nested |
- Field |
- Constr |
- Method

- Detail:
- Field |
- Constr |
- Method


main.ml2db.al.utility.globaldistance

## Class DistancesCalculator

- java.lang.Object
- - main.ml2db.al.utility.globaldistance.DistancesCalculator

- All Implemented Interfaces:
  :   java.io.Serializable

  ---

    

  ```
  public class DistancesCalculator
  extends java.lang.Object
  implements java.io.Serializable
  ```

  Calculates the distance between two instances. Distances are stored in memory
  for reuse. If no distance function is provided, non-normalised Euclidean
  distance is used as default.

  Version:
  :   8 Jul 2011

  Author:
  :   Luna De Ferrari ldf(at)st-andrews.ac.uk

  See Also:
  :   Serialized Form

- - ### Constructor Summary

    Constructors

    Constructor and Description

    `DistancesCalculator(IdMultiLabelInstances instances,
    java.lang.String storageFileName)`

    The distances are not saved to file.

    `DistancesCalculator(IdMultiLabelInstances instances,
    java.lang.String storageFileName,
    boolean writeToFile)`

    `DistancesCalculator(IdMultiLabelInstances instances,
    java.lang.String storageFileName,
    boolean writeToFile,
    weka.core.NormalizableDistance distanceFunction)`
  - ### Method Summary

    Methods

    | Modifier and Type | Method and Description |
    | --- | --- |
    | `void` | `calculateAllDistances()` Fill storage with all possible distances (before serialization) |
    | `java.util.Vector<java.lang.String>` | `getDistance(weka.core.Instance first, weka.core.Instance second)` |
    | `weka.core.DistanceFunction` | `getDistanceFunction()` |
    | `java.lang.String` | `getDistancesFileName()` |
    | `java.lang.String` | `getFilePath()` |
    | `IdMultiLabelInstances` | `getInstances()` |
    | `DistancesStorage` | `getStorage()` |
    | `uk.ac.standrews.utils.main.stringutils.StringPool` | `getStringPool()` |
    | `boolean` | `isWriteToFile()` |
    | `static void` | `main(java.lang.String[] args)` |
    | `void` | `setDebug(boolean m_debug)` |
    | `void` | `setDistanceFunction(mulan.data.MultiLabelInstances instances, weka.core.NormalizableDistance distanceFunction)` Generates a default Eucidean distance if the distance function is null |
    | `void` | `setFilePath(java.lang.String m_filePath)` |

    - ### Methods inherited from class java.lang.Object

      `equals, getClass, hashCode, notify, notifyAll, toString, wait, wait, wait`

- - ### Constructor Detail


    - #### DistancesCalculator

      ```
      public DistancesCalculator(IdMultiLabelInstances instances,
                         java.lang.String storageFileName)
      ```

      The distances are not saved to file.

      Parameters:
      :   `instances` -
      :   `storageFileName` - a short string to customise the name of the file storing the
          distances (for example the arff file id )


    - #### DistancesCalculator

      ```
      public DistancesCalculator(IdMultiLabelInstances instances,
                         java.lang.String storageFileName,
                         boolean writeToFile)
      ```

      Parameters:
      :   `writeToFile` - whether to write the distances to file
      :   `instances` -
      :   `storageFileName` - a short string to customise the name of the file storing the
          distances (for example the arff file id )


    - #### DistancesCalculator

      ```
      public DistancesCalculator(IdMultiLabelInstances instances,
                         java.lang.String storageFileName,
                         boolean writeToFile,
                         weka.core.NormalizableDistance distanceFunction)
      ```
  - ### Method Detail


    - #### calculateAllDistances

      ```
      public void calculateAllDistances()
      ```

      Fill storage with all possible distances (before serialization)


    - #### getDistance

      ```
      public java.util.Vector<java.lang.String> getDistance(weka.core.Instance first,
                                                   weka.core.Instance second)
      ```

      Parameters:
      :   `first` -
      :   `second` -

      Returns:
      :   a vector with first attribute set, second attribute set and
          distance (double)


    - #### getDistanceFunction

      ```
      public weka.core.DistanceFunction getDistanceFunction()
      ```


    - #### getDistancesFileName

      ```
      public java.lang.String getDistancesFileName()
      ```


    - #### getFilePath

      ```
      public java.lang.String getFilePath()
      ```


    - #### getInstances

      ```
      public IdMultiLabelInstances getInstances()
      ```


    - #### getStorage

      ```
      public DistancesStorage getStorage()
      ```


    - #### getStringPool

      ```
      public uk.ac.standrews.utils.main.stringutils.StringPool getStringPool()
      ```


    - #### isWriteToFile

      ```
      public boolean isWriteToFile()
      ```


    - #### setDebug

      ```
      public void setDebug(boolean m_debug)
      ```


    - #### setDistanceFunction

      ```
      public void setDistanceFunction(mulan.data.MultiLabelInstances instances,
                             weka.core.NormalizableDistance distanceFunction)
      ```

      Generates a default Eucidean distance if the distance function is null

      Parameters:
      :   `instances` -
      :   `distanceFunction` -


    - #### setFilePath

      ```
      public void setFilePath(java.lang.String m_filePath)
      ```


    - #### main

      ```
      public static void main(java.lang.String[] args)
      ```


- Overview
- Package
- Class
- Use
- Tree
- Deprecated
- Index
- Help

- Prev Class
- Next Class

- Frames
- No Frames

- All Classes

- Summary:
- Nested |
- Field |
- Constr |
- Method

- Detail:
- Field |
- Constr |
- Method
